# Morphogenesis, Structure, and Chemical Composition of Paiaguás Grass Under Different Nitrogen Doses and Deferment Periods

**DOI:** 10.3390/plants15030341

**Published:** 2026-01-23

**Authors:** Armando Alves de Carvalho, Antonio Leandro Chaves Gurgel, Miguel Arcanjo Moreira Filho, Marcos Jácome de Araújo, Tairon Pannunzio Dias-Silva, Sheila Vilarindo de Sousa, Romilda Rodrigues do Nascimento, Luís Carlos Vinhas Ítavo, Rayanne Amorim Ferreira, Janice Maria dos Santos, Edy Vitoria Fonseca Martins, Auanny Jeniffer de Oliveira Silva, Gelson dos Santos Difante

**Affiliations:** 1 Professora Cinobelina Elvas Campus, Federal University of Piauí, Bom Jesus 64900-000, Brazil; armandocarvalho1995@gmail.com (A.A.d.C.); moreirafilhoma@gmail.com (M.A.M.F.); jacome@ufpi.edu.br (M.J.d.A.); pannunzio@ufpi.edu.br (T.P.D.-S.); sheilavilarindo@gmail.com (S.V.d.S.); rayanneaf99@gmail.com (R.A.F.); janycesantos222@gmail.com (J.M.d.S.); edy29813@ufpi.edu.br (E.V.F.M.); auannyjeniffer9311@gmail.com (A.J.d.O.S.); 2 Bom Jesus Technical College, Federal University of Piauí, Bom Jesus 64900-000, Brazil; romildarn01@ufpi.edu.br; 3 Faculty of Veterinary Medicine and Animal Science, Federal University of Mato Grosso do Sul, Campo Grande 79070-900, Brazil; luis.itavo@ufms.br (L.C.V.Í.); gelson.difante@ufms.br (G.d.S.D.)

**Keywords:** *Brachiaria brizantha*, forage production, nitrogen fertilization, stockpiled pasture, tropical grasslands

## Abstract

The study evaluated the effects of nitrogen fertilization on the morphogenetic, structural, productive, and nutritional characteristics of *Brachiaria brizantha* cv. Paiaguás subjected to two stockpiling periods in a pot experiment. The experiment was conducted using a randomized block design in a 4 × 2 factorial arrangement, with four nitrogen doses (0, 25, 50, and 75 mg N dm^−3^, applied as urea) and two stockpiling periods (80 and 120 days). Increasing nitrogen doses promoted linear increases in leaf appearance, elongation, and senescence rates, as well as tiller population density, while reducing phyllochron and leaf lifespan. Forage mass increased linearly with nitrogen, ranging from 96.25 to 113.00 g of dry matter per pot, and leaf blade mass showed a similar response. Root mass exhibited a quadratic response, with a maximum estimated value of 49.33 g pot^−1^ at 60.18 mg N dm^−3^, this quadratic equation explained 96% of the variation in the results. No significant interaction was observed between nitrogen doses and stockpiling periods for dry matter, crude protein, mineral matter, or neutral detergent fiber contents. However, nitrogen fertilization increased crude protein content across plant fractions, with leaf crude protein rising from about 70 to over 110 g kg^−1^ dry matter. Nitrogen fertilization at 75 mg N dm^−3^ combined with an 80-day stockpiling period improves canopy structure, forage production, and nutritional quality of Paiaguás grass, highlighting the importance of synchronizing nitrogen supply with deferment duration in deferred pasture management.

## 1. Introduction

Deferred grazing is a management technique that involves the temporary exclusion of a portion of the pasture area to allow forage growth and accumulation, which will later be used for grazing during periods of scarcity [1,2]. It is a technique that is easy to implement and highly feasible, standing out mainly due to its low implementation cost. This practice typically involves closing off approximately 40% of the total pasture area at the beginning of February, which will be used as deferred pasture from May to July; the remaining area is closed in early March for use from August to mid-October [3].

However, growth and development, including senescence, are outcomes inherent to the deferment process [4], since the plant grows under free-growth conditions, resulting in excessive stem elongation and, consequently, forage losses due to senescence [5,6]. These structural changes in the pasture can lead to forage of low nutritional value, especially when the pasture is poorly managed [7,8]. This occurs because, under excessively long deferment periods, a large proportion of tillers complete their phenological cycle, transitioning from the vegetative to the reproductive stage [9].

In this context, reducing deferment periods is recommended to obtain a higher quality. However, such reduction generally results in lower forage mass during the utilization period of the deferred pasture [9]. Nitrogen fertilization therefore emerges as an effective strategy to mitigate this limitation, as it promotes greater forage production even under short deferment periods. This is because nitrogen (N) is the nutrient with the greatest potential to enhance forage yield [9], given that it accelerates tissue turnover in forage grasses by increasing leaf appearance and elongation rates, stem elongation, and leaf senescence [10].

Another relevant aspect for the success of the deferment technique is the selection of the cultivar to be used [11]. BRS Paiaguás is a *Brachiaria brizantha* cultivar released in 2013 as an option for pasture diversification on medium-fertility soils [12]. It was selected based on productivity, vigor, and seed production, and although it does not exhibit resistance to pasture spittlebugs, it has shown high potential for animal production during the dry season, with a high leaf proportion and good nutritive value [13]. A major advantage of the Paiaguás cultivar is its performance during the dry season, when it presents greater forage accumulation with improved nutritive value, resulting in higher weight gain per animal and per unit area [14]. The hardiness, adaptation to dry periods, growth habits, and nutritive value of this *Brachiaria* cultivar justify investigating its responses when subjected to deferment in semiarid regions.

In this context, it is hypothesized that higher nitrogen doses, when combined with shorter deferment periods, may result in forage mass comparable to that obtained in Paiaguás grass pastures subjected to longer deferment periods, but with superior nutritional quality. The objective of this study was to evaluate the effect of nitrogen fertilization on the morphogenetic, structural, and nutritional characteristics of *Brachiaria brizantha* cv. Paiaguás subjected to two deferment periods.

## 2. Results

No significant interaction (*p* > 0.05) was observed between nitrogen (N) doses and deferment period for the morphogenetic and structural variables analyzed. However, a positive linear effect of N doses was detected on leaf appearance rate (LAR), leaf elongation rate (LER), leaf senescence rate (LSR), and tillering population density (TPD), with estimated increases of 0.0004 leaves/tiller/day, 0.006 cm/tiller/day, 0.003 cm/tiller/day, and 0.232 tillers/pot, respectively. In contrast, increasing nitrogen fertilization resulted in a linear reduction of 0.032 days in the phyllochron and 0.189 days in leaf lifespan (Table 1). No significant effect of N doses was observed (*p* > 0.05) on stem elongation rate (SER), number of living leaves (NLL), or final leaf length (FLL).

There was no effect of deferment period on LAR, phyllochron, Leaf lifespan (LL), LSR, or TPD, with mean values of 0.111 leaves/tiller/day, 21.29 days, 15.47 days, 1.441 cm/tiller/day, and 36.22 tillers/pot, respectively. However, higher LER, FLL, and NLL and lower SER were observed in Paiaguás grass deferred for 80 days (Table 2).

No significant interactions were observed between nitrogen doses and the deferment period in the evaluation of productive traits (*p* > 0.05). In addition, nitrogen doses did not influence stem mass, leaf–stem ratio, live–dead ratio, or shoot–root ratio. On the other hand, increasing nitrogen doses resulted in a linear reduction in plant height (*p* = 0.026), with a decrease of 0.115 cm. However, a linear increase was observed in forage mass (β_1_ = 0.218 g/pot), leaf blade mass (β = 0.058 g/pot), and dead material mass (β = 0.100 g/pot). Root mass fitted a quadratic egression model, and the derivative of the equation estimated the maximum root mass (49.33 g/pot) at the nitrogen dose of 60.18 mg dm^−3^ (Table 3). This quadratic equation explained 96% of the variation in the results.

There was no effect of the deferment period on plant height, stem mass, root mass, or the shoot–root ratio. However, greater forage mass and dead material mass were observed, along with lower leaf blade mass, leaf–stem ratio, and live–dead ratio, in palisadegrass deferred for 120 days (Table 4).

Lignin concentration in palisadegrass was influenced by the interaction between nitrogen doses and deferment periods, both in the leaf and stem fractions. For leaf lignin, at 80 days of deferment, the concentrations were not affected by nitrogen doses. However, at 120 days, lignin values decreased with increasing nitrogen fertilization, from 232.09 g/kg dry matter (DM) in the absence of N to 176.51 g/kg DM with 75 kg/ha of N. In the stem fraction, there was a linear reduction in lignin concentration regardless of the deferment period evaluated (Table 5). Furthermore, regardless of plant fraction (leaf or stem) or nitrogen dose, higher lignin concentrations were observed in forage deferred for 120 days.

There was no significant interaction between nitrogen doses and deferment periods for the DM, mineral matter (MM), crude protein (CP), and neutral detergent fiber (NDF) contents in the leaf, stem, and dead material fractions of palisadegrass. Similarly, no significant interaction was observed for lignin content in the dead material fraction (Table 6). A linear increasing effect of nitrogen doses was observed on the DM and CP contents of leaves, stems, and dead material (*p* < 0.05). However, MM contents of these components decreased as nitrogen doses increased (*p* < 0.05; Table 7). On the other hand, increasing nitrogen fertilization resulted in reduced NDF contents (*p* < 0.05) in all components evaluated, as well as reduced lignin content in the dead material fraction (Table 6).

With respect to the deferment period, an increase was observed in the DM and NDF contents of the leaf fraction (*p* < 0.05), the NDF content of the stem fraction (*p* < 0.05), and the DM content of the dead material fraction (*p* < 0.05). Conversely, CP contents decreased in all fractions as the number of deferment days increased (Table 7).

## 3. Discussion

Nitrogen is an essential element for accelerating plant tissue turnover, as it is a component of proteins involved in the synthesis of structural compounds in plants [9]. In the present study, a positive linear effect of N doses was observed on LAR, LER, LSR, and TPD. Conversely, increasing nitrogen fertilization resulted in a linear reduction in phyllochron and leaf lifespan (Table 1). In a pasture under vegetative growth, tiller morphogenesis can be described by the parameters LAR, LER, and leaf lifespan. These genetically determined components are strongly influenced by environmental factors, such as nitrogen supply [15]. This nutrient promotes an increase in leaf appearance, elongation, and senescence rates while simultaneously reducing leaf lifespan and the phyllochron [10]. Additionally, nitrogen stimulates the sprouting of axillary buds, resulting in greater tiller production [10,16].

According to Fagundes et al. [17], nitrogen fertilization increases LAR and LER due to the effect of this nutrient on plant physiology. Nitrogen is responsible for increasing the number of dividing cells, which stimulates the production of new cells and consequently enhances LER. In addition, the effect of nitrogen on TPD can be explained by the increased formation of axillary buds, which may subsequently give rise to new tillers [15].

On the other hand, the increase in N doses did not change SER, NLV, or FLL. Although FLL is influenced by LAR and LER [15], it did not show variation despite the increase in these rates resulting from nitrogen supply. This lack of response may be attributed to the reduction in LL, caused by the greater nitrogen availability. At higher N doses, leaves elongated more rapidly but for a shorter period (lower LL); conversely, at lower doses, elongation was less intense but occurred over a longer period (greater LL), generating a compensatory effect. Finally, the NLV of a forage plant tiller is a genetically determined trait that establishes a constant number of live leaves, showing little variation in response to environmental factors, since leaf expansion and senescence rates tend to balance each other [18].

The deferral period did not affect LAR, phyllochron, LL, LSR, or TP. However, higher LER, lower SER, greater FLL, and a higher NLV were observed in Paiaguás palisadegrass deferred for 80 days (Table 3). Extending the deferral period from 80 to 120 days should be avoided, as the combination of higher LER, NLV, and FLL with lower SER in the pasture deferred for 80 days may result in a canopy structure with a greater proportion of leaves and fewer stems. In addition, the longer leaves observed in the 80-day deferred pasture may modify the leaf area index [19] and, consequently, the amount of light intercepted by the forage canopy [20]. The increase in FLL also influences the ease with which grazing animals harvest forage, considering that cattle remove approximately 50% of the leaf blade in each bite [21]. Therefore, smaller leaves may hinder the animals’ daily intake capacity due to a reduction in bite volume.

Nitrogen fertilization promoted a linear increase in forage mass, leaf blade mass, and dead material mass. Nitrogen fertilization is a management tool commonly used in pastures to enhance the productive capacity of forage plants [9]. This nutrient is considered the “fuel” of plant cells because it intensifies tissue turnover (appearance and death) and can modify the structural and productive characteristics of plants [10]. In this context, the increases observed in forage mass, leaf blade mass, dead material mass, and root mass can be attributed to the effect of nitrogen on the rates of leaf appearance and elongation, resulting in higher tiller population densities and a greater rate of leaf senescence (Table 1).

The increase in nitrogen doses resulted in a linear reduction in plant height. This response can be explained by physiological mechanisms related to the balance between tiller size and tiller density. Taller plants generally have larger tillers but lower tiller density, whereas shorter plants exhibit higher tiller density with smaller tillers [22]. At the physiological level, tillering is regulated by the activation of axillary buds, which is influenced by the leaf appearance rate. However, not every emerging leaf gives rise to a tiller, as this process is affected by hormonal signals, carbohydrate allocation, and nutrient availability, including nitrogen [15]. Elevated nitrogen levels can enhance tiller initiation but may also accelerate leaf expansion and senescence, ultimately reducing stem elongation and plant height [10]. This interplay of hormonal and nutritional signals explains the observed linear reduction in height with increasing nitrogen doses.

Although multiple studies [10,15,17] demonstrate the impact of nitrogen on forage growth, it is important to acknowledge the limitations inherent to pot experiments. Unlike field conditions, pot studies restrict root volume, limit soil buffering capacity, and may alter water and nutrient dynamics, which can affect nitrogen uptake and plant growth patterns. In the field, plants experience more heterogeneous soil conditions, larger rooting volumes, and variable microclimatic factors, which influence nitrogen utilization efficiency and forage yield potential. Therefore, while the present pot experiment provides controlled insights into nitrogen effects and plant responses, the results should be interpreted with caution when extrapolating to field conditions. These differences highlight the critical role of the soil–plant–environment interaction, emphasizing that nitrogen dynamics and growth responses observed in pots may differ quantitatively, and occasionally qualitatively, from those in situ. Future studies under field conditions are recommended to validate and expand upon the findings reported here.

Greater forage mass and dead material mass were observed, along with lower leaf blade mass, leaf-to-stem ratio, and live-to-dead ratio in Paiaguás palisadegrass deferred for 120 days. Extending the deferment period from 80 to 120 days should be avoided because, although it increases total forage mass at the end of the period, this mass is composed mainly of dead material and exhibits a low proportion of live leaves, resulting in a low leaf-to-stem ratio. Pastures deferred for 120 days tend to develop a structural arrangement that hinders forage apprehension and intake, as components such as stems and dead material constitute significant physical barriers to achieving deep bites. Furthermore, the greater presence of dead tissues indicates that forage obtained after 120 days of deferment will exhibit a high degree of lignification and lower digestibility coefficients.

The reduction in leaf lignin concentration in response to nitrogen fertilization, observed only at the 120-day deferment period, can be explained by the interaction between plant maturity and nitrogen availability affecting carbon allocation and cell wall deposition. At longer deferment periods, tissues are more differentiated and lignification is naturally higher [23]. Under these conditions, increased nitrogen availability favors protein synthesis and leaf expansion, leading to a dilution effect and shifting carbon allocation away from secondary cell wall components, such as lignin. In contrast, at the shorter deferment period (80 days), tissues are less mature and lignification is lower, limiting the detectable effect of nitrogen on lignin content [24].

The absence of interactive effects on other indicators, such as crude protein and neutral detergent fiber, can be explained by their different metabolic regulation. Crude protein accumulation is strongly responsive to nitrogen availability regardless of tissue maturity, while neutral detergent fiber reflects structural carbohydrates that are less sensitive to short-term nitrogen fluctuations. This distinction highlights why lignin responds specifically to the interaction of nitrogen and tissue developmental stage, ensuring logical coherence of the observed results.

In the literature, the isolated effect of increasing nitrogen doses on the linear reduction in lignin concentration in deferred *Brachiaria* pastures is not unanimous, as this variable is known to interact with season of the year, deferment period, forage cultivar, and especially plant age [23]. With advanced maturation at 120 days, nitrogen likely acted by promoting tissue renewal, thereby reducing lignification, whereas at 80 days plants were still at developmental stages in which nitrogen exerted a similar influence. Overall, nitrogen fertilization tends to increase forage production without decreasing forage digestibility; another commonly reported effect of N application is the reduction in ash concentration [24]. The use of nitrogen in deferred *Brachiaria* pastures has proven effective in increasing dry matter production even during seasons in which pasture growth is reduced due to low temperatures, limited water availability, and shortened photoperiod. Nitrogen doses of 100 kg/ha applied at the end of summer, combined with deferment periods of 95 days, significantly increased dry matter production throughout the year [25].

Maranhão et al. [26] reported that applying 200 kg of nitrogen per hectare *to Brachiaria decumbens* pastures increased daily dry matter production during the summer, autumn, and winter. In the summer, production rose by 109%, while increases of 17% and 13% were observed in autumn and winter, respectively. The authors concluded that nitrogen fertilization enhanced the seasonal distribution of annual forage production.

Studies conducted over five consecutive years evaluated the effects of increasing nitrogen doses (0, 90, 180, and 270 kg N ha^−1^) on the protein and carbohydrate fractions of *Urochloa brizantha* cv. Marandu pastures. The authors observed that nitrogen fertilization increased crude protein content linearly (from 103 to 173 g kg^−1^ DM) and total digestible nutrients, while reducing neutral detergent fiber. These changes indicate an improvement in forage quality, with greater availability of protein and energy. Nitrogen application at 90 kg ha^−1^ stood out for yielding high levels of crude protein, soluble protein, and TDN, suggesting that this dose is the most appropriate for maximizing forage nutritive value and reducing the need for protein supplementation [27].

Nitrogen fertilization in *Brachiaria* cultivars increases leaf crude protein content; with the application of 200 kg N per hectare, CP content reaches 8.64%, with proportional increases as nitrogen rates rise. This effect is consistent throughout the year and indicates that nitrogen enhances protein metabolism and improves pasture nutritive value. At the same time, reductions in NDF are observed, with 63.87% at the 200 kg N ha^−1^ dose, a response associated with a higher leaf-to-stem ratio and a greater proportion of young tissues in the sward canopy [28].

In studies evaluating nitrogen doses ranging from 0 to 270 kg ha^−1^ year^−1^ in Marandu grass pastures, crude protein content remained around 12%, while neutral detergent fiber decreased to values below 60%, indicating improved forage digestibility. The reduction in NDF, combined with increases in crude protein and dry matter, reinforces that nitrogen fertilization is an efficient tool to intensify forage production and enhance its nutritive value, contributing to more productive and sustainable production systems [29]. According to Dupas et al. [30], NDF content decreased with increasing nitrogen doses, with 175 kg ha^−1^ providing the highest dry matter yields.

Applying high nitrogen doses is an effective strategy to increase production and accelerate growth in deferred pastures without compromising forage quality. Regardless of the deferment period, higher nitrogen rates increase forage mass, leaf blade mass, dead material, and root mass, while also enhancing leaf appearance and elongation rates and tiller lifespan. Thus, when combined with shorter deferment periods, high nitrogen fertilization constitutes an efficient strategy to improve both the productive and the morphogenetic and structural characteristics of deferred pastures.

## 4. Materials and Methods

The experiment was conducted in the Forage and Pasture Sector of the Professora Cinobelina Elvas *campus* (CPCE) of the Federal University of Piauí (UFPI), located in the municipality of Bom Jesus, Piauí, Brazil. The city is situated in the southern region of the state, in the Alto-Médio Gurguéia microregion, at a latitude of 09°04′28″ South and a longitude of 44°21′31″ West, with an altitude of 277 m. According to the Köppen climate classification (1931), the municipality has an Aw climate (tropical climate with a dry winter season).

To minimize the influence of environmental heterogeneity, the pots were randomly distributed within each block. Environmental conditions were continuously monitored: the average daytime temperature ranged from 28 to 35 °C, the nighttime temperature from 20 to 29 °C, the mean relative humidity was 35%, and the lighting was 100% natural, with no supplemental artificial light. The greenhouse used in the experiment was covered with a M24 Tropical diffuser film (7.60 m wide × 0.12 mm thick), suitable for tropical regions, which evenly diffuses light and promotes plant growth. Additionally, the sides of the greenhouse were fitted with a white mosquito net screen.

Before the beginning of the experiment, soil samples were collected from an area of the *campus*, sieved, and analyzed for characterization (Table 8), correction, and fertilization according to the recommendations for the evaluated cultivar. However, the soil characteristics already met the requirements of the Paiaguás cultivar, making the application of soil amendments unnecessary.

According to the World Reference Base for Soil Resources [31], the soil used in the experiment was classified as an Arenosol. This classification is based on its predominantly sandy texture, low organic matter content, and moderate natural fertility, as indicated by the soil physical and chemical analyses. The soil showed no diagnostic horizons associated with highly weathered or clay-enriched soil groups, supporting its classification within the Arenosol reference group.

After analysis, 7 dm^3^ of soil were placed in each pot and irrigated to field capacity. Brachiaria brizantha cv. Paiaguás was sown in early March 2024 with 30 seeds per pot, and thinning was performed on the twentieth day, leaving five plants per pot. To promote uniform initial growth, a pre-application of 50 mg N dm^−3^ as urea (45% N) was applied to all pots. Subsequently, the experimental nitrogen treatments (0, 25, 50, and 75 mg N dm^−3^) and the two deferment periods (80 and 120 days) were applied. The nutrient solution was prepared using water as a solvent, with urea diluted to maintain the same nitrogen proportion across all doses without adding other nutrients, and applied directly to the soil of each experimental unit using a beaker to ensure uniform distribution.

Throughout the experimental period, the pots were kept free of weeds and pests and were irrigated according to the methodology adapted from Gomide and Gomide [32], ensuring that soil moisture did not exceed field capacity. The watering regime followed adaptations of the methodologies proposed by Leão et al. [33] and Staudinger et al. [34]. Water was supplied only when complete leaf blade folding occurred, preventing the plants from reaching the permanent wilting point. This management strategy aimed to simulate the water stress experienced by plants under field conditions during deferment [35].

At the beginning of the evaluations, three tillers per pot were marked to determine their morphogenetic and structural characteristics. The tillers were identified with colored wires and measured weekly using a ruler graduated in centimeters. Measurements were taken for pseudostem height, extended tiller height, and the length of each leaf, which allowed the estimation of the following variables: LAR (leaves tiller^−1^ day^−1^); phyllochron (days leaf^−1^ tiller^−1^); LER (cm tiller^−1^ day^−1^); SER (cm tiller^−1^ day^−1^); FLL (cm tiller^−1^); LSR (cm tiller^−1^ day^−1^);NLL (leaves tiller^−1^); and LL (days), as proposed by Lemaire and Chapman [36]. TPD (tillers pot^−1^) was obtained by counting all tillers present in each experimental unit.

The productive characteristics of Paiaguás grass were determined at the end of the deferment period. Canopy height was measured with a ruler graduated in centimeters at three random points per experimental pot, and each measurement corresponded to the mean curvature height of the leaves around the ruler. Forage mass (FM, g pot^−1^ DM) was estimated by cutting all forage contained in each experimental pot. The samples were weighed, dried in a forced-air oven at 55 °C for 72 h, and reweighed to determine forage dry mass.

To evaluate the morphological components of the forage, a subsample was taken from the material harvested for FM determination. This subsample was separated into leaf (leaf blade), stem (stem + sheath), and dead material. The leaf-to-stem ratio was calculated as the ratio between leaf blade mass (LBM, g pot^−1^ DM) and stem mass (SM, g pot^−1^ DM). The live-to-dead ratio was calculated as the ratio between the sum of leaf blade mass and stem mass and the dead material mass (DMM, g pot^−1^ DM).

The soil and roots from the pots were washed under running water over a set of 1.0 mm and 0.25 mm sieves [37]. The material retained in the smallest sieve was then placed in a forced-air oven at 65 °C until reaching constant weight, for the determination of the shoot-to-root ratio.

The forage morphological component samples were sent to the Animal Nutrition Laboratory of CPCE/UFPI for the determination DM, OM (100 − ash), and CP contents, using methods 930.15, 932.05, and 976.05, respectively [38]. Neutral NDF, and lignin contents were determined following the methodology proposed by Van Soest et al. [39].

The data were subjected to analysis of variance, considering a randomized block design in a 4 × 2 factorial arrangement (four nitrogen doses and two deferment periods), according to the following model:

Y_ijk_ = μ + B_j_ + D_i_ + P_k_ + (D × P)_ik_ + ε_ijk_

where Y_ijk_ = observed value in block j, nitrogen dose i, and deferment period k; μ = overall mean effect; B_j_ = effect of block j; D_i_ = effect of nitrogen dose i (0, 25, 50, and 75 mg dm^−3^); P_k_ = effect of deferment period k (80 and 120 days); (D × P)_ik_ = interaction effect between nitrogen dose and deferment period; ε_ijk_ = random error associated with each observation in block j, dose i, and period k.

When significant by the F-test (α = 0.05), deferment periods were compared using Tukey’s test, and nitrogen dose effects were analyzed by regression. Linear and quadratic models were tested, and the selected model was based on the significance of regression coefficients at the 5% probability level and on the coefficient of determination (*r*^2^).

## 5. Conclusions

The application of 75 mg N dm^−3^, supplied as urea, to Brachiaria brizantha cv. Paiaguás pastures deferred for 80 days, corresponding to an estimated nitrogen fertilization rate of 150 kg N ha^−1^ under field conditions, increases leaf elongation rate, the number of live leaves, and forage mass while reducing stem proportion and improving forage nutritional quality. Additionally, root mass responded quadratically to nitrogen application, with the maximum estimated root mass (49.33 g/pot) occurring at 60.18 mg N dm^−3^, highlighting the importance of balanced nitrogen supply for both above and below-ground growth. These results emphasize the beneficial role of nitrogen fertilization in deferred pastures; however, the magnitude of these responses under field conditions may vary according to soil organic matter content, soil fertility status, and local management practices and should therefore be interpreted with caution.

## Figures and Tables

**Table 1:** Morphogenic and structural characteristics of *Brachiaria brizantha* cv. Paiaguás subjected to different nitrogen doses.

Variables	Nitrogen Doses (mg dm^−3^)				SEM	*p*-Value		Equation	*r* ^2^
	0	25	50	75		L	Q		
LAR (leaves tiller^−1^ day^−1^)	0.08	0.11	0.12	0.12	0.01	0.01	0.12	y = 0.096 + 0.0004x	0.72
Phyllochron (days leaf^−1^ tiller^−1^);	12.24	9.60	10.50	9.29	0.78	0.03	0.37	y = 11.61 − 0.032x	0.60
LER (cm tiller^−1^ day^−1^)	1.98	2.24	2.32	2.43	0.10	0.06	0.47	y = 2.031 + 0.006x	0.93
LL (days)	53.64	41.55	42.39	37.63	2.39	<0.01	0.14	y = 50.88 − 0.189x	0.78
SER (cm tiller^−1^ day^−1^)	0.29	0.25	0.21	0.21	0.04	0.13	0.66	y = 0.24	-
LSR (cm tiller^−1^ day^−1^)	1.27	1.44	1.52	1.52	0.08	0.02	0.28	y = 1.312 + 0.003x	0.83
NLL (leaves tiller^−1^)	4.58	4.75	4.37	4.39	0.29	0.47	0.80	y = 4.52	-
TPD (tillers pot^−1^)	23.87	38.37	40.00	42.63	2.50	<0.001	0.22	y = 27.54 + 0.232x	0.79
FLL (cm tiller^−1^)	17.21	16.65	18.35	16.54	0.79	0.93	0.44	y = 17.19	-

LAR: Leaf appearance rate; LER: Leaf elongation rate; LL: Leaf lifespan; SER: Stem elongation rate; LSR: Leaf senescence rate; NLL: Number of living leaves; TPD: Tillering population density; FLL: Final leaf length; SEM: Standard error of the mean; L: Linear; Q: Quadratic.

**Table 2:** Morphogenic and structural characteristics of *Brachiaria brizantha* cv. Paiaguás subjected to different deferment periods.

Variables	Deferment Period (days)		SEM	*p*-Value
	80	120		
LAR (leaves tiller^−1^ day^−1^)	0.11	0.11	0.01	0.664
Phyllochron (days leaf^−1^ tiller^−1^);	9.99	10.83	0.55	0.293
LER (cm tiller^−1^ day^−1^)	2.55	1.93	0.07	<0.0001
LL (days)	45.66	41.94	1.69	0.135
SER (cm tiller^−1^ day^−1^)	0.2	0.28	0.02	0.048
LSR (cm tiller^−1^ day^−1^)	1.46	1.42	0.05	0.61
NLL (leaves tiller^−1^)	4.98	4.07	0.2	0.005
TPD (tillers pot^−1^)	36.94	35.5	1.77	0.571
FLL (cm tiller^−1^)	18.08	16.3	0.55	0.035

LAR: Leaf appearance rate; LER: Leaf elongation rate; LL: Leaf lifespan; SER: Stem elongation rate; LSR: Leaf senescence rate; NLL: Number of living leaves; TPD: Tillering population density; FLL: Final leaf length; SEM: Standard error of the mean.

**Table 3:** Productive characteristics of Paiaguás grass subjected to different nitrogen doses.

Variables	Nitrogen Doses (mg dm^−3^)				SEM	*p*-Value		Equation	*r^2^*
	0	25	50	75		L	Q		
Height (cm)	39.58	29.96	30.85	29.66	2.72	0.026	0.13	y = 36.85 − 0.115x	0.62
FM (g/pot)	96.25	104.70	109.00	113.00	2.53	<0.01	0.38	y = 97.57 + 0.218x	0.96
LBM (g/pot)	29.75	33.00	34.75	34.00	0.71	<0.01	0.110	y = 30.70 + 0.058x	0.72
SM (g/pot)	35.50	37.00	37.75	40.25	1.63	0.05	0.76	y = 37.62	-
DMM (g/pot)	31.00	34.75	36.50	38.75	1.82	0.01	0.68	y = 31.50 + 0.100x	0.97
L-SR	0.850	0.88	0.94	0.85	0.04	0.78	0.14	y = 0.881	-
L-DR	2.15	2.06	2.07	1.96	0.10	0.24	0.24	y = 2.062	-
RM (g/pot)	39.50	44.00	46.50	42.75	1.71	0.12	0.02	y = 39.28 + 0.296x − 0.003x^2^	0.96
SAR	2.50	2.38	2.38	2.64	0.12	0.46	0.12	y = 2.479	-

FM: forage mass; LBM: leaf blade mass; SM: stem mass; DMM: dead material mass; L-SR: leaf-to-stem ratio; LDR: live-to-dead ratio; RM: root mass; SAR: shoot-to-root ratio; SEM: standard error of the mean; L: linear; Q: quadratic.

**Table 4:** Productive characteristics of Paiaguás grass subjected to different deferment periods.

Variables	Deferment Period (days)		SEM	*p*-Value
	80	120		
Height (cm)	30.96	34.07	1.92	0.2648
Forage mass (g/pot)	103.12	108.37	1.79	0.0493
Leaf blade mass (g/pot)	35.25	30.50	0.50	<0.0001
Stem mass (g/pot)	36.75	38.50	1.15	0.2955
Dead material mass (g/pot)	31.13	39.75	1.29	0.0001
Leaf-to-stem ratio	0.975	0.78	0.03	0.0001
Live-to-dead ratio	2.39	1.80	0.07	0.0001
Root mass (g/pot)	41.75	44.62	1.77	0.1061
Shoot-to-root ratio.	2.49	2.46	0.56	0.8705

SEM: standard error of the mean.

**Table 5:** Breakdown of the interactions between N doses and deferment periods for the lignin concentration of Paiaguás grass.

Deferment Periods	Nitrogen Doses (mg dm^−3^)				*p*-Value		*r* ^2^
	0	25	50	75	L	Q	
Leaf (g/kg DM)							
80	106.40	103.72	116.28	130.10	0.71	0.24	-
120	232.09	200.02	198.21	176.51	0.03	0.46	0.92
*p*-value	0.0001						
Stem (g/kg DM)							
80	70.59	65.20	58.86	54.40	0.04	0.93	0.99
120	309.78	281.49	259.75	254.87	<0.001	0.05	0.99
*p*-value	0.0001						

L: linear; Q: quadratic.

**Table 6:** Chemical composition of Paiaguás grass subjected to different nitrogen doses.

Variables (g/kg DM)	Nitrogen Doses (mg dm^−3^)				SEM	*p*-Value			
	0	25	50	75		L	Q	Equation	*r* ^2^
Leaf									
Dry matter	182.23	210.53	218.77	214.81	9.15	0.02	0.01	y = 190.69 + 0.42x	0.68
Mineral matter	59.55	53.82	44.13	40.86	3.05	<0.001	0.69	y = 59.46 − 0.26x	0.96
Crude protein	39.35	55.02	63.95	74.00	2.50	<0.001	0.27	y = 41.15 + 0.45x	0.98
Neutral detergent fiber	655.03	596.40	594.52	568.48	9.81	<0.001	0.11	y = 642.8 − 1.05x	0.85
Stem									
Dry matter	221.61	218.45	237.34	232.71	9.86	0.25	0.90	-	
Mineral matter	33.12	24.93	30.19	18.98	2.74	0.006	0.59	y = 32.38 − 0.15x	0.59
Crude protein	17.23	25.39	34.45	41.31	2.81	0.05	0.81	y = 17.40 + 0.32x	0.99
Neutral detergent fiber	732.79	703.30	693.07	677.57	7.69	0.02	0.37	y = 728.07 − 0.70x	0.95
Dead material									
Dry matter	291.25	345.53	357.18	409.58	28.71	0.009	0.97	y = 295.89 + 1.46x	0.95
Mineral matter	73.98	51.07	50.08	46.95	6.17	0.007	0.12	y = 67.83 − 0.32x	0.72
Crude protein	10.09	17.71	27.29	32.35	2.08	0.008	0.54	y = 10.40 + 0.30x	0.99
Neutral detergent fiber	701.32	674.72	665.15	661.08	4.87	0.001	0.03	y = 700.75 − 1.19x + 0.009x^2^	0.99
Lignin	284.12	246.54	255.53	243.95	6.05	0.004	0.04	y = 280.76 − 1.22x + 0.01x^2^	0.77

SEM: standard error of the mean; L: linear; Q: quadratic.

**Table 7:** Chemical composition of Paiaguás grass subjected to different deferment periods.

Variables (g/kg DM)	Deferment Period (days)		SEM	*p*-Value
	80	120		
	Leaf			
Dry matter	187.93	225.24	6.47	0.0005
Mineral matter	48.61	50.58	2.15	0.5264
Crude protein	63.43	52.73	1.76	0.0003
Neutral detergent fiber	587.14	620.07	6.94	0.0030
	Stem			
Dry matter	223.11	231.94	6.97	0.3805
Mineral matter	25.26	28.35	1.94	0.2744
Crude protein	36.13	23.06	1.98	0.0001
Neutral detergent fiber	691.08	712.28	5.44	0.0119
	Dead material			
Dry matter	301.38	400.39	20.30	0.0024
Mineral matter	54.31	56.71	4.36	0.7015
Crude protein	32.67	11.04	1.47	0.0000
Neutral detergent fiber	671.33	679.80	3.44	0.0968
Lignin	259.57	255.49	4.28	0.5077

SEM: standard error of the mean.

**Table 8:** Physical and chemical properties of the soil used in the experiment.

K	Ca	Mg	P	Fe	Zn	pH	Al + H	m	V	OM
cmolc dm^−3^			mg/dm^3^			H_2_0	cmolc dm^−3^	%		
0.24	1.4	0.75	15.6	56.3	2.2	5.9	1.5	0.0	61.9	2.3
soil physical properties (g kg^−1^)										
Clay				Silt			Sand			
275.0				44.0			681.0			

m: aluminum saturation; V: base saturation; OM: organic matter. P, K, Fe, and Zn—Mehlich-1 extractor; Ca, Mg, and Al—1 mol L^−1^ KCl extractor; H + Al—calcium acetate extractor at pH 7.0; organic matter—Walkley–Black method.

## Data Availability

The raw data supporting the conclusions of this article will be made available by the authors on request. The data is not publicly available due to institutional data protection policies.
